# The relationship among social support, experienced stigma, psychological distress, and quality of life among tuberculosis patients in China

**DOI:** 10.1038/s41598-021-03811-w

**Published:** 2021-12-20

**Authors:** Xu Chen, Jia Xu, Yunting Chen, Ruiheng Wu, Haoqiang Ji, Yuanping Pan, Yuxin Duan, Meng Sun, Liang Du, Mingcheng Gao, Jiawei Wang, Ling Zhou

**Affiliations:** School of Public Health, Dalian Medical University, 9 Western Section, Lvshun South Street, Lvshunkou District, Dalian, 116044 Liaoning People’s Republic of China

**Keywords:** Tuberculosis, Quality of life

## Abstract

The complex relationships among social support, experienced stigma, psychological distress, and quality of life (QOL) among tuberculosis (TB) patients are insufficiently understood. The purpose of this study was to explore the interrelationships among social support, experienced stigma, psychological distress, and QOL and to examine whether experienced stigma and psychological distress play a mediating role. A cross-sectional survey was conducted between November 2020 and March 2021 in Dalian, Liaoning Province, Northeast China. Data were obtained from 473 TB patients using a structured questionnaire. Structural equation modelling was used to examine the hypothetical model. The research model provided a good fit to the measured data. All research hypotheses were supported: (1) social support, experienced stigma and psychological distress were associated with QOL; (2) experienced stigma fully mediated the effect of social support on psychological distress; (3) psychological distress fully mediated the effect of experienced stigma on QOL; and (4) experienced stigma and psychological distress were sequential mediators between social support and QOL. This study elucidated the pathways linking social support, experienced stigma, and psychological distress to QOL and provides an empirical basis for improving the QOL of TB patients.

## Introduction

Tuberculosis (TB) is a major infectious disease that poses a serious threat to human health and has significant negative social and economic consequences^1,2^. It leads to poor health for millions of people every year and is a major public health problem^1^. In 2019, there were an estimated 10 million new cases of TB worldwide, of which approximately 833,000 were in China, accounting for 8.4% of the global total, ranking third^1^. TB is also the leading cause of death from infectious diseases globally, and approximately 1.41 million people died of TB in 2019, of whom approximately 33,000 died in China, accounting for 2.4% of the global total^1^. The burden of TB remains high in China. Although the suffering caused by TB has been acknowledged for thousands of years, most current TB programs and research have primarily focused on detection, microbiological treatment, prevention, and control, while the quality of life (QOL) of TB patients has been neglected^3,4^.

Although effective anti-TB drugs are available and TB patients have access to effective treatment, TB infectivity, chronic progression, long-term drug treatment over a period of at least 6 months and drug side effects have significantly affected patients’ daily lives, thus affecting their QOL^5–7^. Research has confirmed that TB patients tend to have poor QOL, demonstrating QOL significantly worse than that of the general population^8,9^. The World Health Organization (WHO) defined QOL as an individual’s perception of their position in life within the cultural context and value system in which they live and in relation to their goals, expectations, standards, and concerns^10^. In addition, QOL refers to a person’s subjective assessment of their life’s satisfaction and meaning^11^. QOL can affect treatment adherence in TB patients, while non-adherence to TB treatment is thought to be an important reason for the gap between high financial inputs and poor performance in TB control^12–14^. More importantly, impairments in QOL are associated with poor treatment outcomes, which can increase TB mortality and morbidity and negatively impact TB control^15^. Therefore, it is necessary to explore the factors that influence the QOL of TB patients and to improve the QOL of TB patients. Previous studies have analysed factors associated with QOL. They found that sex, age, education level, marital status, occupational status, monthly income, drug side effects, comorbidities, body mass index (BMI), type of TB, phase of treatment, stigma, depressive symptoms, and social support were associated with QOL^2,6,16,17^.

Social support refers to the amount of perceived and practical care received from family, friends and/or the community^18^. Previous studies have shown that social support affects the QOL of TB patients^9,19^. Patients with adequate social support from family, friends and community are likely to have better QOL^20^. Furthermore, social support was also an important predictor of stigma^21^. Patients with poor social support are more likely to be isolated and alienated, with manifestations such as being denied shared utensils and food by family members and losing their jobs, which may lead to stigma^22,23^. Additionally, good social support will increase life satisfaction and social confidence, enabling patients to adapt to a crisis and reducing the pressure of the patient’s role change, thus also reducing the risk of psychological distress^24^. Previous studies have also demonstrated that perceived social support is associated with psychological distress in TB patients during treatment^25^.

Because TB is transmitted by droplets and is highly contagious, patients with TB often experience great stigma, whether at home, in the workplace or in the community^26^. Studies of patients from a variety of backgrounds have indicated that between 42 and 82% of TB patients report stigma^27,28^. Research has suggested that social stigma may affect life satisfaction in TB patients during and even after treatment^15^, and TB-associated stigma is one of the most important aspects affecting QOL^29^. Stigma disrupts patients’ social interactions with others and reduces social functioning and ability to fulfil daily roles, ultimately endangering patients’ QOL^2^. In addition, studies conducted in rural China and Ethiopia have shown that experienced stigma is significantly associated with psychological distress^30,31^. TB patients who feel stigmatized may less frequently use health services and conceal their illness because of low self-esteem and social isolation. Moreover, studies have reported that TB-associated stigma is associated with psychological stress disorders. These factors can increase the risk of mental health problems, such as psychological distress^21,32,33^.

The main factor that affects the QOL of patients with TB is psychological distress^34^. Studies have indicated that once TB is diagnosed, a wide variety of psychological responses are observed, for example, 51.9% to 81% of TB patients suffer from psychological distress^30,35,36^. Studies have also reported that the presence of mental health problems is the strongest predictor of decreased QOL^8^, and depression is also believed to be an important cause of poor QOL in patients with chronic diseases^37^. Notably, psychosocial burdens may have a greater impact than clinical symptoms in TB patients^34^. Psychological distress may interfere with an individual’s immune response system and affect adherence to anti-TB treatment, which may lead to poor QOL and exacerbate mortality from the disease^38,39^.

**Figure 1:**
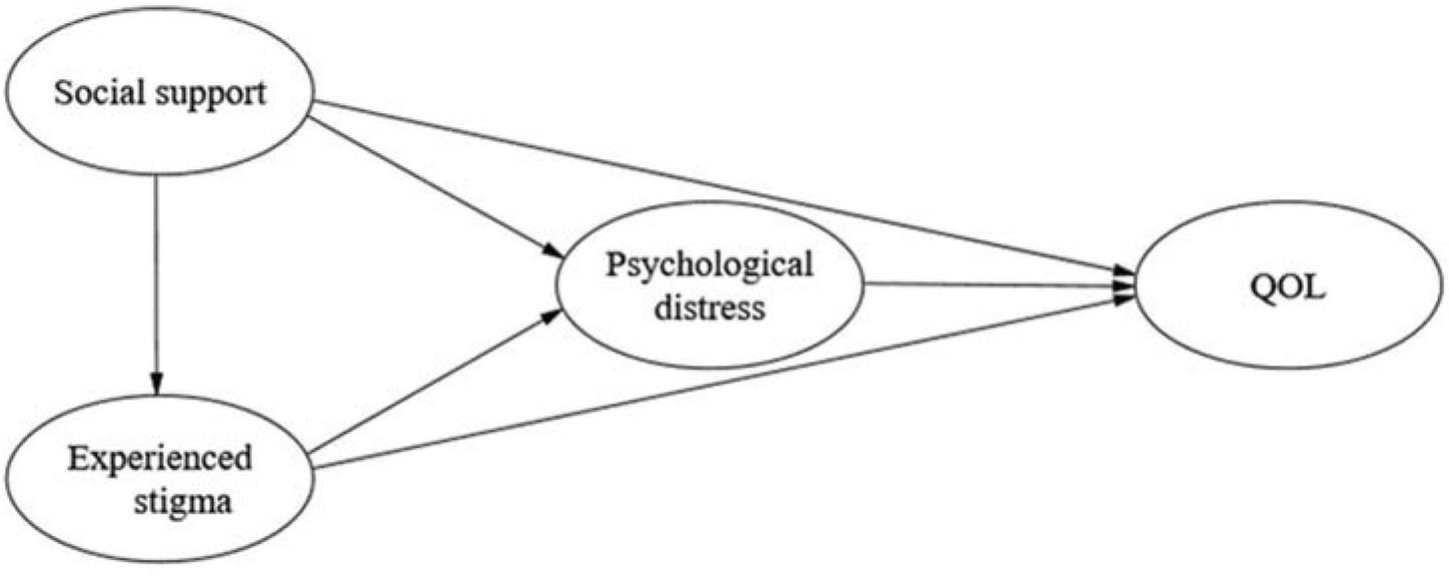
Hypothetical model of relationships among social support, experienced stigma, psychological distress, and QOL. *QOL* quality of life.

QOL of TB patients is generally neglected in existing national TB control programs^17^, and the lack of research on influencing factors of QOL may be one of the key reasons. As mentioned above, previous studies mainly relied on regression analysis and mostly explored only the direct relationships among variables. The pathways reflecting social support, experienced stigma, and psychological distress effects on QOL remain unclear. Without this understanding, it is difficult to determine precisely which variables should be the primary target of QOL priority interventions. Structural equation modelling (SEM), however, aims to decompose the direct and indirect effects of variables, discover the potential and important associations, and produce a more complete picture of causal effect mechanisms to understand the mechanisms and pathways that might explain these relationships^40,41^. In addition, the SEM incorporates measurement errors into the research model, which is more robust than the regression model^42,43^. The use of SEM enables us to untangle the complex relationships among social support, experienced stigma, psychological distress and QOL. Understanding the mechanisms and pathways of the relationship among social support, experienced stigma, psychological distress and QOL can help accurately determine the intervention objectives to improve QOL for TB patients, improve the effectiveness of intervention measures, achieve better clinical management, and ultimately increase the possibility of obtaining the best treatment outcomes and achieving the WHO’s strategy to end TB. Based on the above theory and empirical research results, we proposed a hypothetical model (Fig. 1). As illustrated in Fig. 1, the current study aimed to test the following hypotheses: (1) social support, experienced stigma, and psychological distress are associated with QOL (H1); (2) experienced stigma mediates the relationship between social support and psychological distress (H2); (3) psychological distress mediates the relationship between experienced stigma and QOL (H3); and (4) experienced stigma and psychological distress are sequential mediators from social support to QOL (H4). According to these research hypotheses, we suggest ways to improve the QOL of TB patients.

## Methods

### Study design and setting

A cross-sectional, questionnaire-based survey was carried out between November 2020 and March 2021 at three TB medical institutions in Dalian, Liaoning Province, Northeast China. The three medical institutions were selected based on the number of patients attending, type of patient and location. The first is the only tertiary specialized hospital for TB prevention and control in Dalian and is divided into northern and southern parts located in Ganjingzi District and Pulandian District, respectively. It currently has nearly 500 beds, serving the whole city’s TB patients, especially critically ill TB patients, as the main medical institution for TB patients in Dalian. The other two institutions are TB dispensaries located in Lushunkou District and Zhuanghe City (a county-level city), which serve only local TB patients with a milder instance of the disease.

### Participants

TB patients who attended the selected TB medical institutions between November 2020 and March 2021 were recruited as participants. The inclusion criteria were patients with a definite TB diagnosis according to national TB program guidelines, aged 18 years or older and with a new or relapsed case of TB undergoing treatment. The exclusion criteria were patients with psychosis, communication problems, or difficulty understanding the questionnaire and completion of treatment. A total of 481 patients were recruited and completed a structured questionnaire. Of the 481 questionnaires obtained, eight were excluded due to logical errors or large amounts of missing data. Ultimately, this study included 473 TB patients, with a participation rate of 98.34%.

### Ethics procedure

Ethical approval was provided by the Ethics Committee of Dalian Medical University, Liaoning Province, China. Before participating in the study, each participant was informed of the purpose of the study and how the results would be presented and received guarantees that their personal information would not be disclosed. Each participant voluntarily signed an informed consent form to participate in our study. All methods in our study were conducted in accordance with relevant guidelines and regulations (Declaration of Helsinki).

### Measurement

A structured questionnaire consisting of questions concerning demographic characteristics, treatment status, social support, experienced stigma, psychological distress, and QOL was developed by reading a large amount of relevant literature and consulting experts in related fields. Demographic characteristics included sex, age, marital status and education level. Treatment status included the category of TB treatment, phase of treatment and self-assessed disease severity.

#### Social support

Social support was measured using the Oslo 3-item social support scale, a 3-item questionnaire that is commonly used to assess social support-related issues in clinical and community settings^44^. This questionnaire contains questions that ask patients about the number of people they feel close to and on whom they could count on for serious problems, how much people cared about them and the ease with which they could receive practical help from neighbours. Its overall score ranges from 3 to 14, with a high score indicating a high level of social support. In the current study, the scale’s Cronbach’s α reliability coefficient was 0.718.

#### Experienced stigma

Experienced stigma was assessed using a 9-item stigma questionnaire developed in accordance with Chinese social and cultural contexts^45^. The questionnaire assesses the stigma experienced by patients on the three dimensions of prejudice, discrimination, and rejection. Responses to each item were rated on a 4-point Likert scale, ranging from strongly disagree (= 1) to strongly agree (= 4). The scores of each item were summed to obtain the total score (range 9–36). Higher scores indicate higher levels of stigma experienced by TB patients. The scale showed good reliability and validity, and its Cronbach’s α in this study was 0.946.

#### Psychological distress

The Kessler Psychological Distress Scale (K-10) questionnaire was used to assess psychological distress in TB patients^46^. Numerous studies have demonstrated the reliability and validity of this scale^30^. The scale is composed of 10 items divided among four subscales: nervousness, agitation, fatigue and negative affect^47^. Negative affect includes hopelessness, low mood, sadness and a sense of worthlessness. An example of such an item is “How often did you feel hopeless in the last 30 days?”. The frequency of each item was recorded on a 5-point Likert scale, ranging from none of the time (= 1) to all the time (= 5). The overall score ranged from a low of 10 to a high of 50, indicating an increase in psychological distress. In this study, the scale had high internal consistency (Cronbach’s α = 0.929).

#### QOL

The QOL is an index of the satisfaction levels of the body, spirit, family and social life and the overall evaluation of life^48^. In the current study, a 6-item quality of life scale (QOL-6) developed by Phillips in 2002 was used to measure QOL in TB patients^49^. This scale consists of six items covering physical health, psychological health, economic circumstances, work, family relationships and relationships with nonfamily members. Patients were asked to rate the extent to which the six traits reflected their actual life situation over the past month. Each item was recorded on a 5-point Likert scale, ranging from very poor (= 1) to excellent (= 5). The overall score ranged from 6 to 30, with a high score reflecting good QOL. This scale has been used to assess the QOL of different populations^17,49,50^. In the current study, the scale had acceptable internal consistency (Cronbach’s α = 0.792). QOL was parcelled to produce three categories using the item parcelling method for the final model analysis^51^.

### Statistical analysis

The complete and correct questionnaires were inputted into the database established using EpiData 3.1 software (EpiData Association, Odense, Denmark) by double entry to ensure the accuracy of the data. The data were exported to SPSS 21.0 (IBM Corporation, Armonk, State of New York) for preliminary statistical analysis. Descriptive statistical analysis included the frequency and percentage of classified data and the mean and standard deviation (SD) of continuous data. *T* tests and analysis of variance were used to compare QOL scores among different groups. *Pearson* correlation analysis was used to evaluate bivariate correlations. All comparisons were two-tailed, and *P* < 0.05 was considered statistically significant.

When multiple potential mediating variables and complex relationships were considered in the research model, we used AMOS 23.0 software (IBM Corporation, Armonk, New York, USA) to conduct structural equation modelling (SEM) to test the hypotheses. Confirmatory factor analysis (CFA) was carried out to test the reliability and validity of the constructs and combined with SEM to improve the research model^42^. The maximum likelihood method was used to estimate the parameters. Additionally, the 95% confidence interval (*CI*) was calculated using bootstrapping with 5000 resamples for all effects^52^. The bootstrapping performed was a non-parametric test that does not rely on assumptions of normal distribution, and the effect was considered statistically significant if the 95% *CI* did not include zero. The goodness-of-fit index (GFI), comparative fit index (CFI), Tucker-Lewis index (TLI), standardized root mean square residual (SRMR), and root mean squared error of approximation (RMSEA) were calculated to examine the fit of the model. GFI, CFI and TLI values were greater than 0.900, and SRMR and RMSEA values were less than 0.080, indicating adequate goodness of fit^53^.

## Results

### Participants’ demographic characteristics and treatment status

Among the 473 participants, the mean age was 48.36 (SD = 17.58) years, and most participants (60.04%) were aged 45 years or older. There were more than twice as many male participants (69.13%) as female participants (30.87%). There were slightly more participants with a high school education or above (34.88%) than those with a middle school education (33.19%) or a primary education or below (31.92%). Nearly two-thirds of the participants (65.33%) were married, and only 72 (15.22%) had relapsed. More than half of the patients (59.20%) were in a continuous phase of treatment, and nearly one-third (29.60%) felt that their current condition was severe. Among the respondents, the average QOL score was 20.41 (SD = 3.65). Age, marital status, education level, treatment category, treatment phase and self-assessed severity were significantly associated with QOL (*P* < 0.05) (Table 1).

**Table 1:** Participants’ demographic characteristics and treatment status and their associations with QOL.

Variables	Total n (%)	Quality of life		*P* value
		Mean	SD	
**Sex**				0.391
Male	327 (69.13)	20.32	3.76	
Female	146 (30.87)	20.63	3.38	
**Age (years)**				** < 0.001**
18–30	107 (22.62)	22.11	2.96	
31–44	82 (17.34)	20.93	3.72	
45 or above	284 (60.04)	19.63	3.63	
**Marriage status**				** < 0.001**
Single	116 (24.52)	21.02	3.91	
Married	309 (65.33)	20.61	3.40	
Divorced or widowed	48 (10.15)	17.69	3.46	
**Education level**				** < 0.001**
Primary or below	151 (31.92)	19.01	3.69	
Middle school	157 (33.19)	20.17	3.53	
High school or above	165 (34.88)	21.91	3.14	
**Treatment category**				**0.005**
New	401 (84.78)	20.61	3.62	
Relapse	72 (15.22)	19.31	3.64	
**Treatment phase**				**0.023**
Intensive phase	193 (40.80)	20.86	3.28	
Continuous phase	280 (59.20)	20.11	3.86	
**Self-assessed severity**				** < 0.001**
Mild	333 (70.40)	21.26	3.39	
Severe	140 (29.60)	18.41	3.47	

*SD* standard deviation.

Significant values are given in bold.

### Correlations of the variables

The mean scores of social support, experienced stigma, and psychological distress were 9.71 (SD = 2.27), 18.86 (SD = 7.14), and 19.62 (SD = 7.49), respectively. Social support was negatively correlated with experienced stigma (*r* =  − 0.263, *P* < 0.01) and psychological distress (*r* =  − 0.151, *P* < 0.01) and positively correlated with QOL (*r* = 0.579, *P* < 0.01). In addition, experienced stigma was positively correlated with psychological distress (*r* = 0.453, *P* < 0.01) and negatively correlated with QOL (*r* =  − 0.429, *P* < 0.01). Psychological distress was negatively correlated with QOL (*r* =  − 0.480, *P* < 0.01) (Table 2).

**Table 2:** Descriptive statistics and correlations among study variables.

Variables	Mean ± SD	Social support	Experienced stigma	Psychological distress
Social support	9.71 ± 2.27			
Experienced stigma	18.86 ± 7.14	− 0.263**		
Psychological distress	19.62 ± 7.49	− 0.151**	0.453**	
QOL	20.41 ± 3.65	0.579**	− 0.429**	− 0.480**

*QOL* quality of life, *SD* standard deviation.

All correlations were significant. **P < 0.01.

### Reliability and validity of the constructs

Through factor analysis, the unstandardized estimates of each item were significant (*P* < 0.001), and the standardized factor loadings of each item were > 0.5, which met the physical requirements of factor analysis, indicating that each item has a substantial effect on the measurement of latent variables. The CR value represents the internal consistency of the construct. The higher the CR is, the greater the internal consistency of the tested factors. In this study, all CR values were > 0.7, indicating that the constructs exhibited acceptable internal consistency. Moreover, AVE is the average of the explanatory power of the calculated latent variable to the observed variable. The higher the AVE is, the higher the convergent validity. The value of AVE is recommended to be greater than 0.5. In this study, the AVE ranged from 0.500 to 0.819, which implied that the interpretation degree of latent variables with respect to the observed variables was good and the convergent validity of the constructs was high. The values of $\sqrt{AVE}$s in the diagonal were greater than or slightly lower than the *Pearson* correlation coefficient of other related constructs. This fact indicates that the discriminant validity among factors is significant and that each factor can be well separated. Overall, these constructs exhibited good reliability and validity^54,55^ (Tables 3, 4).

**Table 3:** Reliability analysis of the constructs.

Construct	Item	Parameter significance estimation				Std.	SMC	CR
		Unstd.	S.E.	*t* value	*P*			
Social support	ss1	1.000				0.900	0.810	0.739
	ss2	0.892	0.101	8.839	***	0.661	0.437	
	ss3	0.521	0.065	8.027	***	0.502	0.252	
Experienced stigma	Rejection	1.000				0.926	0.857	0.931
	Prejudice	0.957	0.032	29.510	***	0.885	0.783	
	Discrimination	0.907	0.029	30.802	***	0.904	0.817	
Psychological distress	Nervousness	1.000				0.783	0.613	0.905
	Agitation	1.167	0.061	19.174	***	0.807	0.651	
	Fatigue	1.113	0.057	19.627	***	0.821	0.674	
	Negative affect	1.162	0.051	22.670	***	0.942	0.887	
QOL	qol1	1.000				0.630	0.397	0.795
	qol2	1.180	0.100	11.814	***	0.880	0.774	
	qol3	0.800	0.065	12.377	***	0.732	0.536	

Three items of the Oslo social support scale are labelled ss1, ss2, and ss3. Three categories of the QOL scale are labelled qol1, qol2, and qol3.

*QOL* quality of life, *Unstd.* unstandardized estimate, *S.E.* standard error, *Std.* standardized estimate/factor loading, *SMC* squared multiple correlations, *CR* composite reliability.

***P < 0.001.

**Table 4:** Validity analysis of the constructs.

	AVE	QOL	Psychological distress	Experienced stigma	Social support
QOL	0.569	**0.754**			
Psychological distress	0.706	− 0.494	**0.840**		
Experienced stigma	0.819	− 0.470	0.492	**0.905**	
Social support	0.500	0.778	− 0.174	− 0.326	**0.707**

*QOL* quality of life, *AVE* average of variance extracted.

The bold values on the diagonal in the table are $\sqrt{\mathrm{AVE}}$s, and the values underneath the bold value represent the *Pearson* correlation coefficients between the constructs.

### Fit indices of the overall research model

Table 5 shows the fit indices of the overall research model. To evaluate the fit of the overall model, the following commonly used fitness indices were used: Chi-square test of the model fit = 395.162; degrees of freedom = 125; Chi-square/DF = 3.161; GFI = 0.914; CFI = 0.939; TLI = 0.916; SRMR = 0.072; RMSEA = 0.068. In this context, GFI, CFI and TLI values greater than 0.900 indicate good model fit. SRMR and RMSEA values less than 0.080 suggest adequate model fit. All the fitness indices in the current study met the practical standards or thresholds and reached the ideal level, which indicated that the overall research model fit well^56^ (Table 5).

**Table 5:** Fit indices of the overall research model.

Index	Criteria	Research model	Support or not
Chi-square	Small is better	395.162	Support
DF	Large is better	125	Support
Chi-square/DF	1 < Chi-square/DF < 5	3.161	Support
GFI	> 0.900	0.914	Support
CFI	> 0.900	0.939	Support
TLI	> 0.900	0.916	Support
SRMR	< 0.080	0.072	Support
RMSEA	< 0.080	0.068	Support

*DF* degrees of freedom, *GFI* goodness-of-fit index, *CFI* comparative fit index, *TLI* Tucker-Lewis index, *SRMR* standardized root mean square residual, *RMSEA* root mean squared error of approximation.

### Effect analysis of the research model

**Figure 2:**
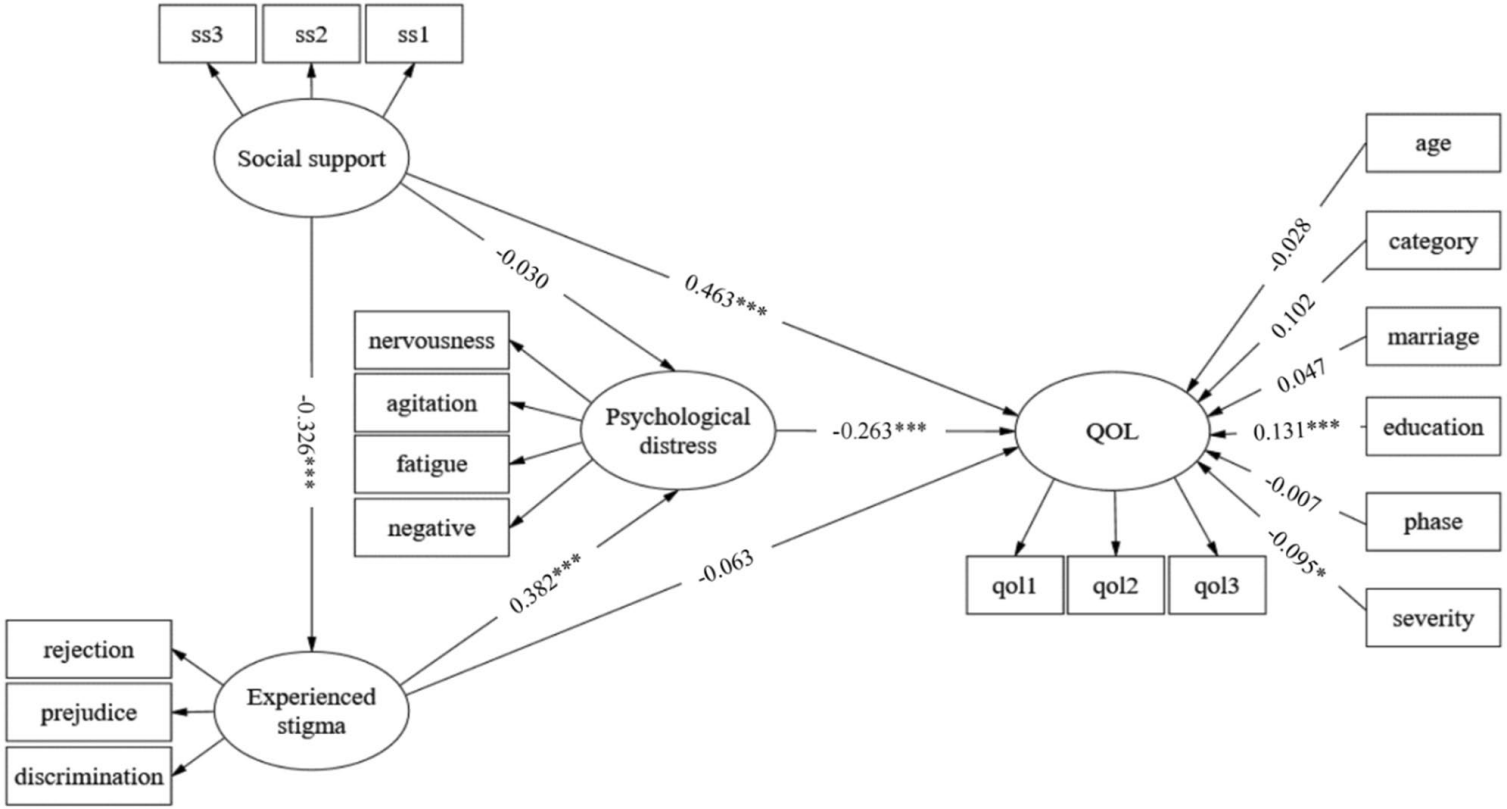
Pathway analysis of the relationships among social support, experienced stigma, psychological distress, and QOL. All the coefficients have been unstandardized in the figure. Three items of the Oslo social support scale are labelled ss1, ss2, and ss3. Three categories of the QOL scale are labelled qol1, qol2, and qol3. Age, treatment category, marriage status, education level, treatment phase and self-assessed severity acted as covariates. ***P < 0.001, *P < 0.05. *QOL* quality of life, *negative* negative affect, *category* treatment category, *marriage* marriage status, *education* education level, *phase* treatment phase, *severity* self-assessed severity. Fit of the model: Chi-square test of the model fit = 395.162; degrees of freedom = 125; GFI = 0.914; CFI = 0.939; TLI = 0.916; SRMR = 0.072; RMSEA = 0.068.

Figure 2 shows the research model with unstandardized path coefficients. Age, marital status, education level, treatment category, treatment phase and self-assessed severity acted as covariates. Education level was positively associated with QOL (*β* = 0.131, *P* < 0.001), while self-assessed severity was negatively associated with QOL among TB patients (*β* =  − 0.095, *P* < 0.05). As shown in Table 6, the total effect of social support on psychological distress was − 0.154 (95% *CI* (− 0.245, − 0.068) and (− 0.243, − 0.067)). Social support significantly predicted psychological distress via experienced stigma (95% *CI* (− 0.187, − 0.075) and (− 0.184, − 0.074)). However, the direct effect of social support on psychological distress was nonsignificant (95% *CI* (− 0.117, 0.052) and (− 0.114, 0.054)). Therefore, experienced stigma fully mediates the effect of social support on psychological distress. The total effect of experienced stigma on QOL was − 0.163 (95% *CI* (− 0.246, − 0.088) and (− 0.249, − 0.090)). Experienced stigma significantly predicted QOL via psychological distress (95% *CI* (− 0.156, − 0.095) and (− 0.156, − 0.059)). However, the direct effect of experienced stigma on QOL was also nonsignificant (95% *CI* (− 0.137, 0.012) and (− 0.137, − 0.011)). Thus, psychological distress fully mediates the effect of experienced stigma on QOL. The total effect of social support on QOL was 0.524 (95% *CI* (0.435, 0.635) and (0.435, 0.635)), and the direct effect was 0.463 (95% *CI* (0.386, 0.562) and (0.384, 0.561)), accounting for 88.36% of the total effect. In addition, social support significantly predicted QOL via the sequential mediation variables of experienced stigma and psychological distress (95% *CI* (0.017, 0.058) and (0.016, 0.057)), whose estimated multiple indirect effect was only 0.033, accounting for 6.30% of the total effect. In sum summary, all the hypotheses were supported (Fig. 2, Table 6).

**Table 6:** Analysis results of mediating variables (5000 bootstrap samples).

Relationships	Point estimation	Product of coefficients		Bootstrapping			
				Bias-corrected 95% *CI*		Percentile 95% *CI*	
		S.E	*Z*	Lower	Upper	Lower	Upper
**Effect from SS to PD**							
Indirect effect							
SS → ES → PD	− 0.124	0.028	− 4.429	− 0.187	− 0.075	− 0.184	− 0.074
Direct effect							
SS → PD	− 0.030	0.044	− 0.682	− 0.117	0.052	− 0.114	0.054
Total effect							
SS → PD	− 0.154	0.045	− 3.422	− 0.245	− 0.068	− 0.243	− 0.067
**Effect from ES to QOL**							
Indirect effect							
ES → PD → QOL	− 0.100	0.025	− 4.000	− 0.156	− 0.059	− 0.156	− 0.059
Direct effect							
ES → QOL	− 0.063	0.038	− 1.658	− 0.137	0.012	− 0.137	0.011
Total effect							
ES → QOL	− 0.163	0.041	− 3.976	− 0.246	− 0.088	− 0.249	− 0.090
**Effect from SS to QOL**							
Indirect effect							
SS → ES → QOL	0.021	0.013	1.615	− 0.002	0.049	− 0.003	0.047
SS → PD → QOL	0.008	0.012	0.667	− 0.013	0.033	− 0.014	0.032
SS → ES → PD → QOL	0.033	0.011	3.000	0.017	0.058	0.016	0.057
Direct effect							
SS → QOL	0.463	0.045	10.289	0.386	0.562	0.384	0.561
Total effect							
SS → QOL	0.524	0.052	10.077	0.435	0.635	0.435	0.635

*SS* social support, *ES* experienced stigma, *PD* psychological distress, *QOL* quality of life, *S.E.* standard error, *CI* confidence interval.

## Discussion

Patients with TB often have symptoms such as cough, chest pain, low fever, fatigue, and loss of appetite. In addition, the treatment of TB is a complex and lengthy process, requiring many medications and a long period of treatment. These factors significantly affect the QOL of patients^17^. However, to date, the complex relationships among social support, experienced stigma, psychological distress, and QOL in patients with TB have not been fully explored. To our knowledge, this study was the first to use SEM to explore the interrelationships among social support, experienced stigma, psychological distress, and QOL and to examine whether experienced stigma and psychological distress play mediating roles.

In the current study, factor analysis indicated that each construct displayed good reliability and validity, which further verified the stable structure of the scale in TB patients and provides a basis for future studies to measure the social support, experienced stigma, psychological distress, and QOL of TB patients. More importantly, the fitness indices exhibited good model fit, indicating that our proposed research model is reasonable and provides key information for improving the QOL of TB patients. Moreover, this study found that education level was associated with QOL in terms of demographic characteristics. Previous studies have also demonstrated that education level is an important predictor of QOL, such that a higher education level has a positive effect on the QOL of TB patients^2,9^. Patients with higher levels of education contribute to greater knowledge about TB from the outside world. Knowledge of TB can improve health-related behaviors such as taking anti-TB drugs on time and seeking care in a timely manner^57^. This will contribute to the effective control of the disease and reduce the patients’ stigma, thus reducing psychological distress and improving the QOL. However, patients with low levels of education may lack a correct understanding of TB. This often leads to doubt about the ability to cure TB and reduced self-efficacy^58^. Patients with low self-efficacy also have stronger stigma experience^59^, which increases the risk of psychological distress and affects the QOL of patients. This study also found that patients with perceived severe illness had worse QOL than those with mild illness. Previous studies have also demonstrated that worse physical symptoms are associated with lower physical health-related QOL and higher mental health-related QOL among TB patients^60^. Understandably, patients with more severe disease have more complex clinical conditions and longer treatment times, as well as increased patient concerns, which may be particularly damaging to QOL. In addition, it is understandable that the more severe the illness are, the more obvious the symptoms. Obvious symptoms, especially a prolonged cough, may lead to a greater degree of accidental disclosure of the illness. This will have a negative impact on access to social support, increase stigma and psychological distress and threaten QOL.

The results also showed that social support demonstrated a significant, direct effect on the QOL of patients with TB. Social support helps improve patients’ QOL^20^, which has also been found in studies on patients with traumatic brain injury^61^. A possible explanation is that patients who receive adequate social support might have improved health outcomes. Moreover, consistent with previous studies, stigma was a predictor of QOL^62^. Stigma can damage patients’ self-esteem and self-efficacy, lead to patients’ isolation from society and self-concealment, and ultimately endanger patients’ QOL^63^. In addition, psychological distress exerted a direct effect on QOL in our study. Studies have reported that untreated depression is independently associated with poorer QOL^39^. Another study also demonstrated that mental distress had a significant effect on QOL^6^. Patients with psychological distress were less likely to adhere to treatment regimens, thus eliminating the chance of successful treatment, impairing their function, and reducing QOL^39,64^.

Previous research has demonstrated that patients who receive an adequate amount of social support are likely to have the best mental health outcomes^20^. Our results suggest that social support can also have an indirect negative effect on psychological distress through experienced stigma. Sufficient social support can increase patients’ self-esteem and make patients more likely to be diagnosed in a timely manner and to comply with treatment, thus reducing the occurrence of psychological distress^65,66^. In addition, our results confirmed that psychological distress moderated the relationship between experienced stigma and QOL. It is not difficult to understand that the experience of stigma will lead to patients’ feelings of inferiority, lack of confidence and low emotional well-being, which threaten patients’ emotions and cause psychological distress, thus affecting their QOL^33^. Our results also indicated that experienced stigma and psychological distress are sequential mediators from social support to QOL, a relationship that has not been demonstrated in previous studies. However, the findings seem logical because patients with better social support have more emotional and financial resources, which makes them face less discrimination and stress, and are likely to use drugs with fewer side effects; thus, they may have improved QOL^65,67^.

In the current study, SEM was used to test the mediating variables. In epidemiological studies, the assessment of mediation has been widely used to open up the “black box”, allowing us to discern complex relationships between variables^68^. In practice, understanding the interrelationships among social support, experienced stigma, psychological distress and QOL provides an opportunity to intervene effectively in QOL among patients with TB, and it allows for interventions to be tailored to these specific pathways. Specifically, interventions aimed at improving the QOL of TB patients should focus on increasing social support for patients. At the same time, the role of experienced stigma and psychological distress should also be understood and addressed. Given that experienced stigma and psychological distress mediated the effect of social support on QOL in TB patients, interventions should be combined with measures to eliminate stigma and reduce psychological distress. This was essential for improving the QOL of TB patients. Previous studies have demonstrated that family functions and doctor-patient communication are the most important sources of social support for patients^69^. The attitude of family members has an important influence on TB patients. There are widespread psychological burdens among TB patients, such as lack of confidence in a cure and fear of treatment failure^70^. Constant encouragement from and care by family members can increase patients’ confidence and their feelings of being taken care of. Therefore, family members can be educated and trained to provide better support for patients. Doctors also play an important role in the treatment of TB, and a good doctor-patient relationship is the fundamental factor to ensure the normal operation of the treatment process. It is necessary to require medical staff to establish the concept of patient-centred service and to show respect and humanistic care in the process of medical service delivery^71^. It is also important to provide more financial support for patients. Although the country has established some free TB treatment policies, some items, such as the cost of expensive adjuvant drugs, are not included in the free package. Previous studies have also found that TB clubs, composed of health workers and TB patients, have been successful in reducing stigma among TB patients^72^. In addition, community awareness and patient education may contribute significantly to a reduction in stigma^73^.

This study has several limitations that need to be addressed in future studies. First, although SEM was applied, the causal relationship between the variables could not be inferred due to the cross-sectional nature of the data. Therefore, longitudinal studies are needed to validate the current findings. Second, the study sample only included TB patients from Dalian, Liaoning Province, Northeast China, which limited the ability to generalize the results to individuals from other regions with different social and cultural backgrounds. Future research should expand the study area to determine the suitability of our study model. Additionally, the study was limited to TB patients who already had access to health care, while those who did not seek any care were not recruited. The latter are probably the most marginalized and affected by TB, and their participation could enrich our findings. The study also did not include healthy people as controls. Therefore, the results may not capture the impact of TB on patients alone. Finally, only quantitative analysis was conducted in this study, and data were collected through patient self-reports. Patients may hide certain facts, which may cause our results to be underestimated. Extensive interviews and qualitative analysis are needed for a more comprehensive assessment.

## Conclusion

This study empirically explored the interrelationships among social support, experienced stigma, psychological distress, and QOL and tested whether experienced stigma and psychological distress played a mediating role. Using the SEM method, we found that (1) social support, experienced stigma and psychological distress affect the QOL of patients with TB; (2) experienced stigma mediates the relationship between social support and psychological distress; (3) psychological distress mediates the relationship between experienced stigma and QOL; and (4) experienced stigma and psychological distress are sequential mediators from social support to QOL. Understanding and managing the QOL of TB patients may lead to better outcomes, and the results of this study provide useful information to help TB patients achieve better QOL.

## Data Availability

The datasets generated and/or analysed during the current study are available from the corresponding author on reasonable request.
